# A WR3-NH_2_-loaded polysaccharide hydrogel with antibacterial, anti-inflammatory, and pro-healing properties for enhanced wound healing

**DOI:** 10.1016/j.mtbio.2025.102701

**Published:** 2025-12-19

**Authors:** Zhizhi Chen, Chao Li, Lei Wang, Ying Luo, Yahan Yang, Qinqin Han, Jinyang Zhang, Yaoqiang Shi, Yi Sun, Yuzhu Song

**Affiliations:** a Research Center of Molecular Medicine of Yunnan Province, Faculty of Life Science and Technology, Kunming University of Science and Technology, Kunming, Yunnan, 650500, China; b Institute of Basic and Clinical Medicine, The First People's Hospital of Yunnan Province, The Affiliated Hospital of Kunming University of Science and Technology, Kunming, Yunnan, 650032, China

**Keywords:** Hydrogel, Antimicrobial peptide, WR3-NH_2_, Wound healing, Antimicrobial activity

## Abstract

Wound healing is a complex process often compromised by infection, excessive inflammation, and impaired tissue regeneration. In this study, the antimicrobial peptide WR3-NH_2_ loaded multifunctional polysaccharide hydrogel (SGHC-WR) was developed. This hydrogel demonstrated favorable physicochemical properties, including porosity, injectability, degradability, and sustained release capability, while maintaining stability under storage conditions. *In vitro* studies indicated that WR3-NH_2_ significantly enhanced the proliferation and migration of HUVECs, HFF, and HaCaT cells, while also regulating cytokine secretion and modulating the MAPK signaling pathway to mitigate inflammation. SGHC-WR exhibited strong antimicrobial activity against both Gram-positive and Gram-negative bacteria, including resistant strains, with low hemolysis rates and excellent cytocompatibility. *In vivo* evaluations using a full-thickness skin wound model in mice revealed that SGHC-WR accelerated wound closure, enhanced angiogenesis, promoted collagen deposition, and facilitated tissue remodeling. Histological analysis confirmed the regeneration of well-structured epidermis and hair follicles, while biosafety assessments showed no pathological damage in major organs and no dermal irritation following repeated application. Collectively, these findings demonstrate that SGHC-WR creates an optimized wound healing microenvironment by integrating antibacterial, anti-inflammatory, and regenerative functions. This work highlights SGHC-WR as a promising therapeutic biomaterial for advanced wound management and offers new insights into the synergistic application of antimicrobial peptides and polysaccharide hydrogels in regenerative medicine.

## Highlights

• WR3-NH_2_ hydrogel offers a multifunctional platform for wound healing.
• Integrates antibacterial, anti-inflammatory, and pro-regenerative effects.
• Accelerates wound closure and enhances angiogenesis and tissue remodeling.
• Demonstrates excellent biosafety and strong potential for clinical translation.

## Graphical abstract

**Figure:**
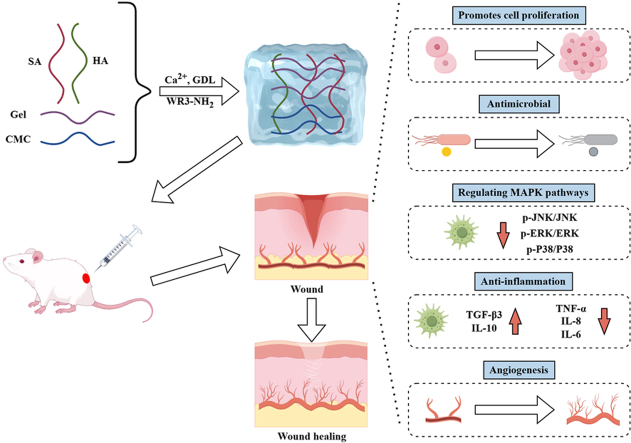

## 1 Introduction

Wound healing is a highly dynamic and complex biological process that encompasses multiple overlapping phases, including hemostasis, inflammation, proliferation, and tissue remodeling [1,2]. Effective wound management is paramount in clinical practice, as traumatic injuries, burns, surgical incisions, and chronic wounds pose a significant global health burden [3]. Delayed or impaired wound healing not only prolongs hospitalization and increases healthcare costs but also leads to severe complications, such as infection, scarring, and even disability [4]. Consequently, the development of advanced wound dressings that can accelerate tissue regeneration while preventing infection has become a pressing clinical need. Traditional wound dressings, such as gauze and cotton pads, primarily provide physical coverage and protection but fail to create a favorable microenvironment for tissue repair [5,6]. In recent years, hydrogels composed of natural or synthetic polymers have garnered increasing attention due to their unique physicochemical properties, including high water content, flexibility, and the ability to maintain a moist environment conducive to healing [[7], [8], [9]]. Moreover, hydrogels offer opportunities for the incorporation of bioactive agents, enabling the design of multifunctional dressings [10,11]. However, conventional hydrogels often lack intrinsic antimicrobial properties and are limited in their ability to modulate inflammation or promote cellular regeneration, thereby restricting their therapeutic efficacy [12].

Antimicrobial peptides (AMPs) are vital components of the innate immune system, exhibiting broad-spectrum antimicrobial activity against both Gram-positive and Gram-negative bacteria, including multidrug-resistant strains [[13], [14], [15], [16]]. In addition to their bactericidal properties, AMPs play a role in regulating immune responses, mitigating excessive inflammation, and promoting angiogenesis and tissue regeneration, thereby making them highly promising candidates for wound therapy [17]. However, the clinical application of AMPs encounters significant challenges: they are prone to enzymatic degradation, possess short half-lives *in vivo*, and may induce cytotoxicity or hemolysis at elevated concentrations [18,19]. Consequently, an effective controlled release system is essential to stabilize AMPs, extend their activity, and minimize adverse effects [20]. Hydrogels derived from natural polysaccharides, such as sodium alginate (SA), hyaluronic acid (HA), and carboxymethyl chitosan (CMC), have been extensively investigated as carriers for AMPs [21,22]. These polymers are biocompatible, biodegradable, and capable of forming porous three-dimensional networks that facilitate drug loading and controlled release [23,24]. Furthermore, their inherent properties can mimic the extracellular matrix, providing structural support for cell proliferation and migration [25,26]. Although several AMP-loaded hydrogel systems have been proposed, most studies focus on singular functions, such as antibacterial activity or moisture retention [2,27]. Comprehensive studies systematically investigating the synergistic effects of AMPs and hydrogels on cell proliferation, inflammation regulation, angiogenesis, and tissue remodeling are still lacking.

To address these gaps, we designed and synthesized the AMP WR3-NH_2_ reported in our previous study [14], which was identified as an AMP derivative from Cathelicidin-DM [28] with potent broad-spectrum antibacterial and anti-inflammatory activities, exhibiting high safety and significant *in vivo* efficacy. To improve its stability and biocompatibility, WR3-NH_2_ was incorporated into a polysaccharide-based hydrogel system (SGHC-WR), composed of SA, HA, gelatin, and CMC. SGHC-WR exhibits favorable physicochemical properties, including injectability, elasticity, degradability, and a porous architecture that supports the controlled release of WR3-NH_2_. In this study, we systematically evaluated the biological performance of the SGHC-WR hydrogels at multiple levels. *In vitro* experiments were conducted to assess its effects on cell proliferation, migration, cytokine secretion, and regulation of the MAPK signaling pathway, as well as its antimicrobial activity and biocompatibility. *In vivo*, a full-thickness skin wound model in mice was used to evaluate wound healing efficacy, anti-inflammatory activity, angiogenesis, collagen deposition, and long-term safety. By combining the antimicrobial and immunomodulatory functions of WR3-NH_2_ with the favorable structural and physicochemical properties of the hydrogel, we confirmed that the SGHC-WR hydrogels can accelerate wound healing, reduce inflammation, prevent bacterial infection, and minimize scar formation. Overall, this work aims to develop a multifunctional wound dressing that integrates antimicrobial, anti-inflammatory, pro-healing, and biosafe features. Our findings not only provide new insights into the synergistic effects of AMPs and polysaccharide hydrogels but also propose a promising strategy for the design of advanced biomaterials in clinical wound management.

## 2 Materials and methods

### 2.1 Synthesis of WR3-NH_2_

WR3-NH_2_ (WRKPCKGWRCWLKRW-NH_2_) was synthesized by Wuhan Dangang Biotechnology Co., Ltd., exhibiting a molecular weight of 2088.45 Da and a purity exceeding 95 %, as confirmed by mass spectrometry and high-performance liquid chromatography analyses.

### 2.2 Cell proliferation assay

Human umbilical vein endothelial cells (HUVECs), human skin fibroblasts (HFF), and human immortalized keratinocytes (HaCaT) were obtained from the Molecular Diagnostics Center of Yunnan Province. The Cell Counting Kit-8 (CCK-8) assay (Bioss, Beijing, China) was employed to evaluate the effect of WR3-NH_2_ on the proliferation of HUVECs, HaCaT, and HFF cells. Cell suspensions were counted using a hemocytometer and adjusted to a concentration of 5.5 × 10^4^ cells/mL. A total of 90 μL cell suspension was added to each well of a 96-well plate, and the cells were incubated at 37 °C in a 5 % CO_2_ incubator for 24 h. Subsequently, WR3-NH_2_ (0, 2.5, 5, 10, and 20 μg/mL) was added, and the cells were incubated for an additional 18 h. Following this incubation, 10 μL CCK-8 reagent was added to each well, and the plate was incubated in the dark for 2 h. Absorbance was measured at 450 nm, and the cell viability was calculated according to the kit's specifications.

### 2.3 Cell migration assay

The cell scratch assay was employed to investigate the effects of WR3-NH_2_ on the migration of HUVECs and HaCaT cells. Prior to the experiment, three lines were marked on the bottom of a 6-well plate. The cell suspension was adjusted to 1 × 10^5^ cells/mL, and 3 mL was added to each well. The cells were incubated at 37 °C in a 5 % CO_2_ incubator until they reached 90 % confluence. Subsequently, the culture medium was removed, and the wells were washed once with PBS. A sterile 200 μL pipette tip was utilized to create vertical scratches along the pre-marked lines. The wells were then washed twice with PBS, and serum-free medium containing WR3-NH_2_ solution (10 μg/mL) was added. For the control group, an equal volume of serum-free medium was added. Cell migration was photographed at 0, 12, 24, 36, and 48 h to document the progress of migration. ImageJ software was employed to measure the area of the scratch, and the scratch recovery rate was calculated using the following formula: scratch recovery rate (%) =(S_0_-S_T_)/S_0_ × 100 %, where S_0_ represents the scratch area at 0 h, and S_T_ denotes the scratch area at various time points.

### 2.4 Cytokine and MAPK signaling pathway detection

ELISA was employed to investigate the effect of WR3-NH_2_ on cytokine expression in a RAW 264.7 (inflammation model cell). Initially, the safe concentration of WR3-NH_2_ was established using the CCK-8 assay. RAW 264.7 cell suspension was adjusted to 1 × 10^5^ cells/mL, and 3 mL of cell suspension was added to the 6-well plate for 24 h incubation. Then, the supernatant was removed, and the cells were washed twice with PBS. Serum-free medium was then added, and both the model and experimental groups were treated with LPS (1 μg/mL) for 6 h, while the control group remained untreated. In the experimental groups, WR3-NH_2_ was administered at final concentrations of 2.5, 5, 10, and 20 μg/mL, and the cells were cultured for an additional 24 h. Subsequently, 1 mL cell supernatant was collected from each well and centrifuged at 4 °C, 4000 rpm for 10 min. The supernatant was then transferred to a new 1.5 mL centrifuge tube, and cytokine expression levels were quantified using an ELISA kit (Enzyme-linked, Jiangsu, China). Simultaneously, the 6-well plates were washed three times with PBS, and 250 μL RIPA lysis buffer (Beyotime Biotechnology, Shanghai, China) was added to each well. The plates were incubated on ice for 30 min, and the lysed cells were scraped off with a cell scraper and transferred to the 1.5 mL centrifuge tubes. The samples were then centrifuged at 4 °C, 12,000 rpm for 15 min, and the supernatants were collected. Protein concentrations were determined using a BCA Protein Assay Kit (Beyotime Biotechnology, Shanghai, China), and the protein samples were adjusted to the same concentration. After the addition of 5 × loading buffer, the samples were heated at 95 °C for 15 min to prepare for Western Blot analysis. The antibodies used in Western Blot analysis for MAPK signaling pathway detection were purchased from Cell Signaling Technology (Danvers, MA, USA).

### 2.5 Preparation of SGHC-WR hydrogel

A series of solutions were prepared, including 1 % SA, 2 % HA, 1 % gelatin (Gel, containing 0.15 % CaCO_3_), and 5 % CMC. Subsequently, 1 % SA, 2 % HA, and 1 % Gel were mixed and stirred in equal proportions (1:1:1), followed by the addition of 0.75 % D-gluconolactone (GDL), the final mixture was designated as SGH. Then, SGH and CMC were combined in varying proportions to create SGHC. Specifically, SGHC2.5 consisted of SGH and CMC in a ratio of 2.5:1, while SGHC3 comprised SGH and CMC in a ratio of 3:1. Finally, WR3-NH_2_ (100 μg/mL) was incorporated into SGHC to yield SGHC-WR. All reagents were purchased from Macklin (Shanghai, China).

### 2.6 Microscopic structure analysis of SGHC-WR

Scanning electron microscopy (SEM) was employed to investigate the microstructure of SGHC hydrogels and the SGHC-WR hydrogels. The prepared hydrogels underwent freeze-drying for 36 h, followed by conductive treatment through vacuum gold sputtering. Subsequently, the treated hydrogel samples were affixed to the SEM sample holder using a sample clamp, allowing for the observation of the hydrogel's microstructure.

### 2.7 Rheological properties testing

The SGHC and SGHC-WR hydrogels were placed in a rheometer (Waters DHR-2, Massachusetts, USA) respectively to assess their storage modulus (G′) and loss modulus (G″) in both oscillatory and rotational modes. In the oscillatory mode, the instrument parameters are set as follows: a fixed strain of 1 % and a frequency of 1 Hz were applied over a time scan of 30 min. In the rotational mode, the viscosity and shear stress of the hydrogels were measured as a function of shear rate, with a shear rate scan range from 1 S^−1^ to 100 S^−1^, while keeping other parameters.

### 2.8 *In vitro* degradation study

A 1.5 mL centrifuge tube was weighed, after which 200 mg of SGHC or SGHC-WR hydrogels were added and the total weight was recorded as the initial value (day 0). The tubes were then kept at room temperature, and the samples were re-weighed on days 2, 4, and 6 to monitor weight changes over time. The degradation rate was calculated using the following formula: Degradation rate (%) = [(W0-Wt)/W0]∗100 %, W0 = weight on day 0, Wt = weight on day 2, 4, or 6.

### 2.9 *In vitro* antimicrobial/killing activity assay

*Staphylococcus aureus* (ATCC 25923), *Escherichia coli* (ATCC 25922), and multidrug-resistant *Staphylococcus aureus* (ATCC 29213) were obtained from the Molecular Diagnostic Center of Yunnan Province. Single colonies were selected and cultured in Muller-Hinton (MH) medium, and the bacterial suspension was adjusted to 1 × 10^5^ CFU/mL using a 1.5 × McFarland turbidity standard. Subsequently, 500 μL SGHC and SGHC-WR hydrogels were added to 2 mL centrifuge tubes, and equal volume of MH medium as the negative control group. To each tube, 500 μL of the adjusted bacterial suspension was added, and the tubes were incubated at 37 °C, 180 rpm. Absorbance at 600 nm was measured at 0, 6, 12, and 24 h. Finally, after 24 h, the bacterial suspension was diluted 1000-fold, plated, and bacterial colony growth was counted.

### 2.10 Stability analysis

Given that the WR3-NH_2_ demonstrates significant antimicrobial activity [14], its stability in SGHC-WR hydrogels over time was evaluated. The SGHC-WR hydrogels, which contains WR3-NH_2_ at a concentration of 100 μg/mL, was prepared and stored at both room temperature and 4 °C. The antimicrobial activity was assessed every other day. For the testing method, a single colony of *S. aureus* (ATCC 25923) in good growth condition was selected for culture and the bacterial suspension was adjusted to 1 × 10^5^ CFU/mL. Subsequently, 50 μL SGHC-WR hydrogels and WR3-NH_2_ (100 μg/mL) stored at room temperature or 4 °C were added to each well of a 96-well plate, equal volume of MH medium as the negative control group. Finally, 50 μL of the adjusted bacterial suspension was added to each well, and the plate was sealed and incubated at 37 °C for 18 h. The absorbance at 600 nm was measured to evaluate the antimicrobial activity.

### 2.11 Hemolytic activity assay

A total of 3 mL blood was collected from healthy kunming mice, and immediately centrifuged at 4 °C, 3000 rpm for 10 min, after which the supernatant was discarded. The red blood cells were resuspended and washed with physiological saline until the supernatant became clear to prepared into a 2 % suspension. Transferred 360 μL red blood cell 2 % suspension to a 0.5 mL centrifuge tube, followed by the addition of 40 μL of the hydrogel sample. The WR3-NH_2_ concentration in the SGHC-WR was 100 μg/mL, and thus, 100 μg/mL WR3-NH_2_ was utilized as the control in the experiment. Negative and positive control groups were established by adding equal volumes of PBS and 1 % Triton X-100 (Servicebio, Wuhan, China), respectively. Each group was tested in quintuplicate. The samples were incubated at 37 °C for 1 h and then centrifuged at 1000 rpm for 15 min. A volume of 100 μL of the supernatant was collected, and the absorbance at 570 nm was measured. The hemolysis rate was calculated using the following formula: Hemolysis rate (%) =(A_2_-A_1_)/(A_3_-A_1_) × 100 %, where A_1_ represents the absorbance of the negative control group, A_2_ denotes the absorbance of the experimental group, and A_3_ indicates the absorbance of the positive control group.

### 2.12 Hemostasis function study

Kunming mice (25–30 g) were selected for this experiment. The mice were anesthetized via intraperitoneal injection of pentobarbital sodium, and their livers were extracted. A piece of known weight filter paper was placed beneath the liver. A 0.5 cm wound was created on the liver using surgical scissors at a consistent location. In the experimental groups, 40 μL SGHC, SGHC-WR hydrogel, and WR3-NH_2_ (40 μg per mouse) were applied to the wound. The blank control group received no treatment. Each experimental condition was performed in triplicate, and after 5 min, the filter paper was removed and weighed.

### 2.13 Cytotoxicity analysis

The CCK-8 assay was employed to evaluate the effects of SGHC and SGHC-WR hydrogels on cell viability. A cell suspension (5.5 × 10^4^ cells/mL) was prepared and dispensed into a 96-well plate at a volume of 90 μL per well. Following a 24-h incubation period, 10 μL of the hydrogel sample was introduced. The concentration of WR3-NH_2_ in SGHC-WR was set at 100 μg/mL. Control groups were established, including the hydrogel with medium, medium with cells, and medium alone, and each group was tested in quintuplicate. After the addition of the hydrogel samples, cell viability was assessed every 12 h, with CCK-8 reagent added at 12, 24, 36, and 48 h. The plate was incubated for 2 h, then the absorbance was measured at 450 nm. The relative cell viability was calculated using the following formula, Cell viability (%) =(A_1_-A_2_)/(A_3_-A_4_) × 100 %, where A_1_, A_2_, A_3_, and A_4_ represent the absorbance of medium with cells, medium alone, medium with cells and hydrogel, and medium with hydrogel, respectively.

### 2.14 Skin sensitivity/irritation test

Healthy Kunming mice were selected for the study, and their dorsal fur was shaved using a razor. SGHC, SGHC-WR hydrogels, and WR3-NH_2_ were applied daily to the backs of the mice, while the control group received sterile water. The treatment was administered continuously for 14 days, and photographs were taken to document the skin condition of the mice on day 0 and day 14.

### 2.15 Construction of mouse full-thickness skin wound model

All animal experiments in this study were approved by the Animal Ethics Committee of Kunming University of Science and Technology (approval number: PZWH K2024-0008), in accordance with the university's guidelines for animal experimentation. Male Kunming mice, purchased from Kunming Medical University and aged 4–5 weeks, were utilized in the construction of mouse full-thickness skin wound model. The mice were anesthetized via intraperitoneal injection of pentobarbital sodium at a dosage of 0.15 mL per 10 g of body weight. The fur on the dorsal side of the mice was shaved using an electric hair clipper, and a circular wound with a diameter of 6 mm was created on the back using a skin punch.

The full-thickness skin wound mice were randomly assigned to four groups: Control, SGHC3, SGHC3-WR, and WR3-NH_2_. In the control group, 50 μL of sterile water was administered daily to the wound. In contrast, the other groups received 50 μL of SGHC3, SGHC3-WR hydrogel, and WR3-NH_2_ (40 μg). Wound healing was documented daily through photography, and ImageJ software was employed to measure the wound area. Wound tissues of mice were collected on the 4th, 8th, and 12th days, fixed with 4 % paraformaldehyde, embedded in paraffin, and then frozen-sectioned. Subsequently, H&E staining, Masson staining, CD31 immunofluorescence, and immunohistochemical analysis were performed according to the instructions of the kit. To further confirm the safety of the SGHC and SGHC-WR hydrogels, the organs of the mice were dissected. The heart, liver, spleen, lungs, and kidneys were excised and fixed in 4 % paraformaldehyde. H&E staining was performed to assess any pathological changes in the internal organs of the mice.

### 2.16 Statistical analysis

All experimental data were analyzed using GraphPad Prism 9.4.1. The results were subjected to two-way ANOVA and Dunnett's multiple comparisons test. Error bars in the figures represent the mean standard error (SEM) from experiments with more than three replicates. Statistical significance was denoted as ∗∗∗∗ (p < 0.0001), ∗∗∗ (p < 0.001), ∗∗ (p < 0.01), and ∗ (p < 0.05).

## 3 Results and discussion

### 3.1 WR3-NH_2_ promotes cell proliferation and migration to accelerate wound healing

**Fig. 1:**
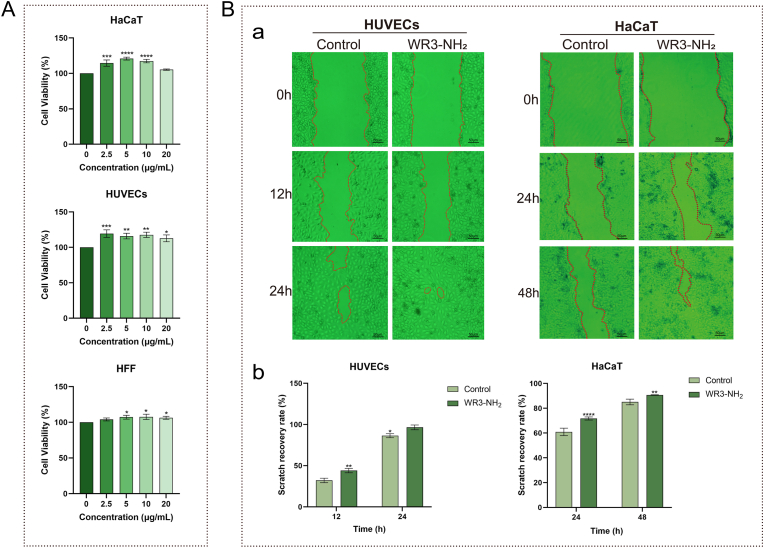
**WR3-NH_2_ promotes cell proliferation and migration to accelerate wound healing. A**: Effect of WR3-NH_2_ on the viability of HUVECs, HFF, and HaCaT cells; **B**: Effect of WR3-NH_2_ on the migration of HUVECs and HaCaT cells. (a) Photographs documenting the effect of WR3-NH_2_ on the migration of HUVECs and HaCaT cells; (b) Scratch recovery rate of HUVECs and HaCaT cells. Error bars in the figures represent the mean standard error (SEM) from experiments with more than three replicates. Statistical significance is indicated as ∗∗∗∗ (p < 0.0001), ∗∗∗ (p < 0.001), ∗∗ (p < 0.01), ∗ (p < 0.05).

WR3-NH_2_ significantly promoted the proliferation of HUVECs, HaCaT, and HFF cells, as well as the migration of HUVECs and HaCaT cells. The results (Fig. 1A) indicated that WR3-NH_2_ exhibited notable pro-proliferative activity on HUVECs and HaCaT cells at 2.5 μg/mL. When the concentration of WR3-NH_2_ was increased to 5 and 10 μg/mL, it demonstrated significant pro-proliferative activity across all three cell types. However, at a concentration of 20 μg/mL, WR3-NH_2_ resulted in a decrease in cellular activity of HUVECs and HaCaT, which may be attributed to the rapid proliferation induced by the high concentration of WR3-NH_2_, leading to nutrient depletion and reduced growth space, ultimately affecting cell viability [29]. Further cell scratch assays (Fig. 1B) revealed that WR3-NH_2_ also significantly enhanced the migration of HUVECs and HaCaT cells. Because these two cell types exhibit different proliferation and migration rates, the observation time points were adjusted accordingly. HUVECs were examined at 12 and 24 h, whereas HaCaT cells were assessed at 24 and 48 h to ensure that meaningful differences in wound closure could be captured for each cell type. After 24 h, the HUVECs scratches were nearly completely healed, with a recovery rate of 96.5 %. In HaCaT cells, the recovery rate of scratches in the WR3-NH_2_ group at 24 h was 71.7 %, which was also significantly higher than the control group's rate of 60.9 %. Although the proliferation rate of HaCaT cells was slightly slower than that of HUVECs, WR3-NH_2_ still exhibited substantial pro-migratory activity at 48 h. These findings demonstrated that WR3-NH_2_ can effectively promote the proliferation and migration of HUVECs and HaCaT cells, potentially accelerating the wound healing process.

### 3.2 WR3-NH_2_ promotes wound healing through cytokine regulation and the MAPK pathway

**Fig. 2:**
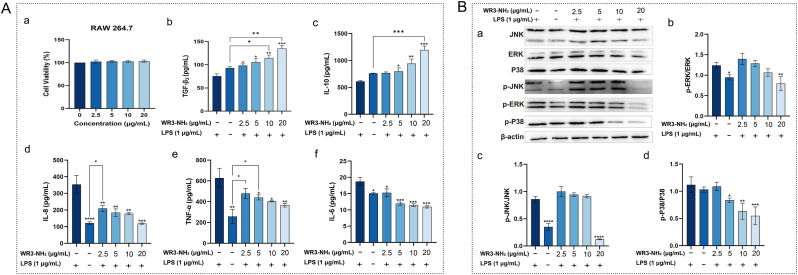
**WR3-NH_2_ promotes wound healing through cytokine regulation and the MAPK pathway. A**: Effects of WR3-NH_2_ on RAW 264.7 cell activity and cytokine secretion in the LPS-induced inflammatory model of the cell. (a) Effects of different concentrations of WR3-NH_2_ on RAW 264.7 cell activity. (b–f) Effects of different concentrations of WR3-NH_2_ on cytokine secretion in the LPS-induced inflammatory model of RAW 264.7 cells. **B**: Effects of WR3-NH_2_ on the MAPK signaling pathway. Error bars in the figures represent the mean standard error (SEM) from experiments with more than three replicates. Statistical significance is indicated as ∗∗∗∗ (p < 0.0001), ∗∗∗ (p < 0.001), ∗∗ (p < 0.01), ∗ (p < 0.05).

WR3-NH_2_ can promote wound healing by regulating cytokine and MAPK signaling pathways. Compared to the LPS model group, WR3-NH_2_ was able to promote the concentration-dependent secretion of TGF-β3 (Fig. 2A–b), an important indicator for assessing whether a wound heals without scarring [[30], [31], [32]]. Normal wounds secrete more TGF-β3, suggesting that WR3-NH_2_ has the potential to promote scarless healing. Additionally, WR3-NH_2_ enhanced the secretion of IL-10 (Fig. 2A–c) while decreasing the levels of TNF-α, IL-8, and IL-6 (Fig. 2A), indicating its anti-inflammatory activity. Further studies demonstrated that WR3-NH_2_ exerts its anti-inflammatory effects by regulating the MAPK signaling pathway (Fig. 2B), which plays a crucial role in inflammation and consists of three major branches: JNK, ERK, and P38. At a concentration of 20 μg/mL, WR3-NH_2_ was able to down-regulate the expression of JNK, ERK, and P38 and inhibit their phosphorylation. Notably, the inhibition of ERK and P38 phosphorylation by WR3-NH_2_ exhibited a weak concentration dependence, while the regulation of JNK phosphorylation showed a strong effect only at 20 μg/mL. Western blot results further validated the anti-inflammatory activity of WR3-NH_2_, suggesting that it may promote wound healing by modulating wound inflammation.

### 3.3 Preparation and characterization of SGHC-WR hydrogels

**Fig. 3:**
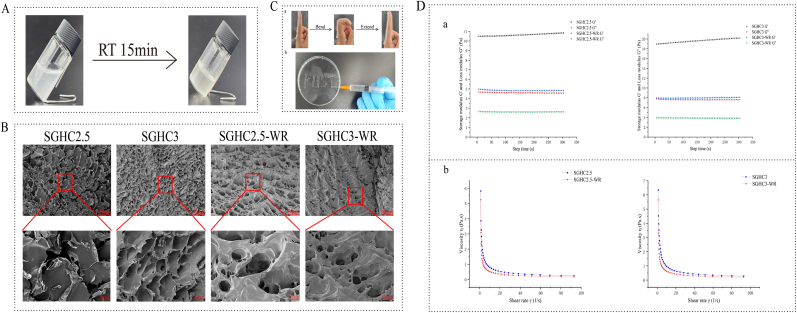
**Preparation and characterization of SGHC-WR hydrogels. A**: Preparation of SGHC-WR hydrogel. **B**: Micromorphology of SGHC/SGHC-WR hydrogel. **C**: Flexibility and injectability test of SGHC-WR hydrogel. **D**: Rheological test of SGHC/SGHC-WR hydrogel. (a) Time scan of fixed strain and frequency in oscillation mode; (b) Logarithmic change of shear rate in rotation mode.

In this study, the SGHC hydrogels were developed with wound healing capabilities using natural polymers, specifically SA and HA, with Ca^2+^ serving as a cross-linking agent [33]. The SGHC hydrogels were formed by allowing it to sit at room temperature for 15 min (Fig. 3A). SEM were employed to compare the microstructures of the SGHC hydrogels and the SGHC-WR hydrogels (Fig. 3B). The results indicated that the SGHC3 hydrogel exhibited a more sparse and porous structure with smaller pore sizes compared to SGHC2.5. Upon the addition of WR3-NH_2_ to SGHC hydrogels, the morphology of the hydrogel altered significantly, becoming more three-dimensional and looser. Specifically, the SGHC2.5-WR hydrogel demonstrated a greater number of pores but a more disorganized arrangement, whereas the SGHC3-WR hydrogel had a relatively smaller number of pores with a more neat and orderly arrangement. These observations in conjunction with rheological tests (Fig. 3D), suggest that the incorporation of WR3-NH_2_ modified the structure of the original hydrogel system, enhancing its elasticity and looseness. This transformation may be attributed to the formation of a preliminary three-dimensional network structure involving WR3-NH_2_, SA, HA, and CMC through electrostatic attraction. Given that cationic AMPs are positively charged and these polysaccharides molecules contain numerous negatively charged carboxyl groups, stable binding is achieved via electrostatic interactions [34]. Additionally, the disulfide bond in WR3-NH_2_ is cleaved to form a sulfhydryl group (-SH) under reducing conditions and subsequently re-forms the disulfide bond through oxidation, thereby facilitating dynamic cross-linking. This process enhances the stability and elasticity of the network structure, promoting the formation of the porous architecture.

Interestingly, SGHC-WR hydrogels were poured into molds to create flakes, which exhibited notable flexibility (Fig. 3C–a). This characteristic enhances the biocompatibility of the SGHC-WR hydrogels for medical and biomedical applications, effectively minimizing trauma or discomfort during interactions with biological organisms. Furthermore, the softness and elasticity of the SGHC-WR hydrogels closely mimic human tissues, increasing their applicability in the fields of bionics and tissue engineering. Additionally, Fig. 3C–b demonstrates the injectability of the SGHC-WR hydrogel, enabling direct injection into the target site for localized treatment. This characteristic facilitates the precise release of drugs, growth factors, or other therapeutic agents directly into damaged tissues or focal areas. Such targeted treatments minimize exposure to systemic medications and create opportunities for personalized treatment regimens. In the material characterization, dynamic mechanical analysis results (Fig. 3D) indicated that G' > G″ for both SGHC2.5 and SGHC3 hydrogels, suggesting that both hydrogels possess good elasticity. However, the lower G′ values also indicate a reduced crosslink density, contributing to their softness and suitability for injection applications. Specifically, the G′ value of SGHC3 was slightly lower than that of SGHC2.5, attributable to its lower crosslink density resulting from a reduced CMC content. The addition of the WR3-NH_2_ to the hydrogel resulted in the decreased G′ and G″ values, indicating that the WR3-NH_2_ diluted the hydrogel system and enhanced its fluidity. The softness and injectability of these hydrogels were further corroborated by the viscosity test presented in Fig. 3D.

### 3.4 Biosafety and compatibility of SGHC-WR hydrogels

**Fig. 4:**
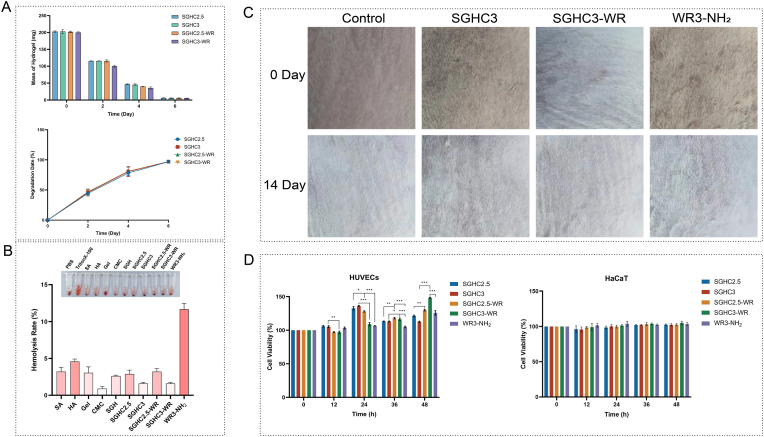
**Biosafety and compatibility of SGHC-WR hydrogels. A**: Mass change and degradation rate of SGHC/SGHC-WR hydrogel. **B**: Hemolytic activity of SGHC-WR hydrogel and its components. The hemolysis of PBS is exactly 0 % and Triton X-100 is exactly 100 % by definition. **C**: Skin sensitivity/irritation test of SGHC3/SGHC3-WR hydrogel, pictures of the back skin of mice before and 14 days after the hydrogel was applied. **D**: Cytotoxicity of SGHC/SGHC-WR hydrogel. Error bars in the figures represent the mean standard error (SEM) from experiments with more than three replicates. Statistical significance is indicated as ∗∗∗∗ (p < 0.0001), ∗∗∗ (p < 0.001), ∗∗ (p < 0.01), ∗ (p < 0.05).

In this study, the potential of SGHC and SGHC-WR hydrogels as wound dressings were explored, focusing on their degradability, biocompatibility, and effects on cell viability. SGHC hydrogels demonstrate a favorable degradability (Fig. 4A), allowing them to gradually degrade upon contact with wounds. This property reduces the frequency of dressing changes and associated pain for patients, which is particularly significant for burns, chronic wounds, or post-surgical treatments [35]. As the hydrogel degrades, it releases medications or bioactive substances, such as AMPs or growth factors, which promote cell growth and tissue regeneration [36]. Additionally, degradable dressings mitigate the risk of infection associated with prolonged wear, as they do not provide an environment conducive to bacterial proliferation [37]. The degradation properties of various hydrogels can be evaluated by measuring changes in their mass [38]. Fig. 4A demonstrated that all four hydrogels were nearly completely degraded on day 6, with degradation rates exceeding 97 %, as evidenced by the overlap of the folded lines in the graphs, suggesting similar degradation capabilities. In the biocompatibility test, the interaction between hydrogels and red blood cell were evaluated, with the hemolysis results presented in Fig. 4B. The SGHC and SGHC-WR hydrogels demonstrated low hemolysis rates of 2.89 % and 1.6 %, respectively, indicating excellent biocompatibility. In contrast, WR3-NH_2_ exhibited a high hemolysis rate of 11.68 %. However, when encapsulated within the hydrogel, this rate was significantly reduced, suggesting that the hydrogel effectively mitigated the direct contact between WR3-NH_2_ and red blood cell, thereby decreasing the immune response and the associated risk of inflammation due to hemolysis. Regarding dermal compatibility, the non-irritating nature of the hydrogels is crucial for their safe application. The hydrogel was applied to the dorsal skin of mice for 14 consecutive days and observed no irritation reactions (Fig. 4C), such as redness or inflammation, which indicating the safety of these hydrogels for skin application. The experimental mice maintained good health, further corroborating the safety of SGHC and SGHC-WR hydrogels.

Furthermore, the effects of SGHC and SGHC-WR hydrogels on the viability of HUVECs and HaCaT cells were assessed by the CCK-8 method. The results (Fig. 4D) showed that at all measured time points (12, 24, 36, and 48 h), the viability of both HUVECs and HaCaT cells was not adversely affected by any of the hydrogel formulations. At 24 h, both SGHC and SGHC-WR hydrogels promoted HUVEC proliferation, whereas a gradual decrease in viability was observed after 36 h, likely due to nutrient depletion in the culture environment rather than hydrogel-induced cytotoxicity. By 48 h, the SGHC3-WR group exhibited the highest HUVEC viability, indicating that the encapsulation and slow release of WR3-NH_2_ enhanced its pro-proliferative effect. In contrast, HaCaT cell viability remained generally stable across all time points. In summary, the SGHC and SGHC-WR hydrogels demonstrated a good degradability and biocompatibility, offering advantages as green and efficient wound dressings, particularly in enhancing cell proliferation and reducing immune responses. Notably, the loading of WR3-NH_2_ into the hydrogel did not diminish its bioactivity, but rather provided greater flexibility and safety for clinical applications.

### 3.5 Antimicrobial activity and hemostatic activity

**Fig. 5:**
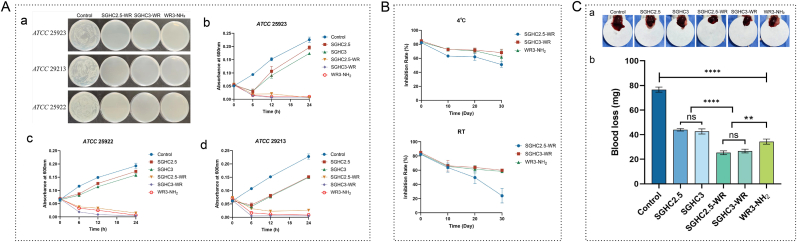
**Antimicrobial activity and hemostatic activity. A:** Antibacterial activity of SGHC/SGHC-WR hydrogel. (a) Plate coating of SGHC-WR hydrogel after co-culture with various strains for 24 h; (b) Inhibition of *S. aureus*; (c) Inhibition of *S. aureus* (MDR); (d) Inhibition of *E. coli*. **B**: Stability test of SGHC-WR hydrogel at 4 °C and room temperature. **C**: Hemostatic property of SGHC/SGHC-WR hydrogel. (a) Photographs were taken to record the bleeding of mouse kidneys; (b) The amount of bleeding in the kidneys of mice in each group. Error bars in the figures represent the mean standard error (SEM) from experiments with more than three replicates. Statistical significance is indicated as ∗∗∗∗ (p < 0.0001), ∗∗∗ (p < 0.001), ∗∗ (p < 0.01), ∗ (p < 0.05).

A comprehensive evaluation of the antimicrobial activity and hemostatic properties of SGHC and SGHC-WR hydrogels were conducted. The incorporation of WR3-NH_2_ was intended to enhance the antimicrobial capacity of the SGHC hydrogel. To assess this effect, Gram-positive bacteria (ATCC 25923, ATCC 29213) and the Gram-negative strain ATCC 25922 were selected for antimicrobial testing. By measuring the absorbance of the bacterial solution at 600 nm every 6 h, the inhibitory effects of the hydrogel on these bacterial strains were observed. The results (Fig. 5A) indicated that WR3-NH_2_ exhibited a slightly greater inhibitory effect on gram-positive bacteria, a trend also observed with the SGHC hydrogel. During the initial 6 h, SGHC showed significantly less inhibition of ATCC 25922 than the other two Gram-positive strains, suggesting a stronger inhibitory effect on Gram-positive bacteria. However, due to the limited number of strains tested, further verification of this conclusion is warranted. Overall, the SGHC hydrogel primarily inhibited bacterial growth rather than directly killing the bacteria. In contrast, the incorporation of WR3-NH_2_ into the SGHC hydrogel significantly improved its antimicrobial performance, making it comparable to WR3-NH_2_ alone, and exhibited a pronounced bactericidal effect within 6 h. In our previous research, WR3-NH_2_ functions as a membrane-disruptive AMP with supplementary intracellular action and immunoregulatory capability [14], supporting its potential as a promising therapeutic candidate. To further confirm its bactericidal efficacy, the hydrogel and bacterial solutions were co-cultivated for 24 h and subsequently plated the samples to observe colony growth. The results (Fig. 5A–a) indicated no colony growth in the WR3-NH_2_ and SGHC3-WR hydrogel groups, while a limited number of colonies were observed in the SGHC2.5-WR hydrogel group on the ATCC 29213 plates. This phenomenon may be attributed to the higher viscosity of SGHC2.5, resulting from its elevated CMC content, which could hinder the release of AMPs and provide a carbon source conducive to colony growth. Considering that ATCC 29213 is a multidrug-resistant strain with significant adaptive capabilities, this may further compromise the bactericidal effect of SGHC2.5-WR. To assess the stability of the hydrogels, their activity under various storage conditions were examined, which is vital for ensuring long-term efficacy [39]. In biomedical and engineering applications, the active stability of hydrogels is essential as it guarantees their physical and chemical properties remain consistent over extended periods, thereby preserving their efficacy. Room temperature and 4 °C were selected as storage conditions and utilized the antimicrobial activity of the hydrogels as a stability indicator. The results (Fig. 5B) demonstrated that the SGHC-WR hydrogel exhibits excellent stability in antimicrobial activity, with SGHC3-WR retaining 60 % of its antimicrobial efficacy after 30 days of storage. This stability study not only ensures predictable and reproducible performance of hydrogels in medical and research contexts but also reduces the frequency and long-term costs associated with replacements, thereby ensuring their efficacy and safety across diverse applications.

In the hemostatic performance evaluation, the hemostasis efficacy of SGHC and SGHC-WR hydrogels were investigated in treating liver wounds in mice. As Fig. 5C shows both WR3-NH_2_, SGHC, and SGHC-WR hydrogels possess hemostatic activity. However, no significant difference was observed between SGHC2.5 and SGHC3. When WR3-NH_2_ was incorporated into the hydrogel, the hemostasis effects of both components were synergistic, resulting in enhanced wound hemostasis. The bleeding was assessed by weighing filter paper sheets placed beneath the liver of the mice. In comparison to the control group, the bleeding was significantly reduced in the SGHC, SGHC-WR, and WR3-NH_2_ groups (Fig. 5C), indicating that both SGHC and SGHC-WR hydrogels possess commendable hemostatic properties. Although the blood loss in the SGHC2.5-WR group was slightly lower than that in the SGHC3-WR group, this difference was not statistically significant. It may be attributed to the higher CMC content in SGHC2.5, which increased the viscosity and consequently enhanced wound adhesion, accelerating hemostasis. Overall, the SGHC3-WR hydrogel demonstrated robust antimicrobial and hemostatic capabilities, rendering it suitable for use in medical dressings. It has the potential to reduce the risks of infection and bleeding while improving healing efficiency in trauma management and surgical procedures. In the realm of medical devices, this hydrogel not only provides lubrication and protection for instruments but also effectively inhibits bacterial growth. The exceptional performance underscores its potential for a wide array of clinical and everyday applications.

### 3.6 Evaluation of wound healing effect of SGHC-WR hydrogel

**Fig. 6:**
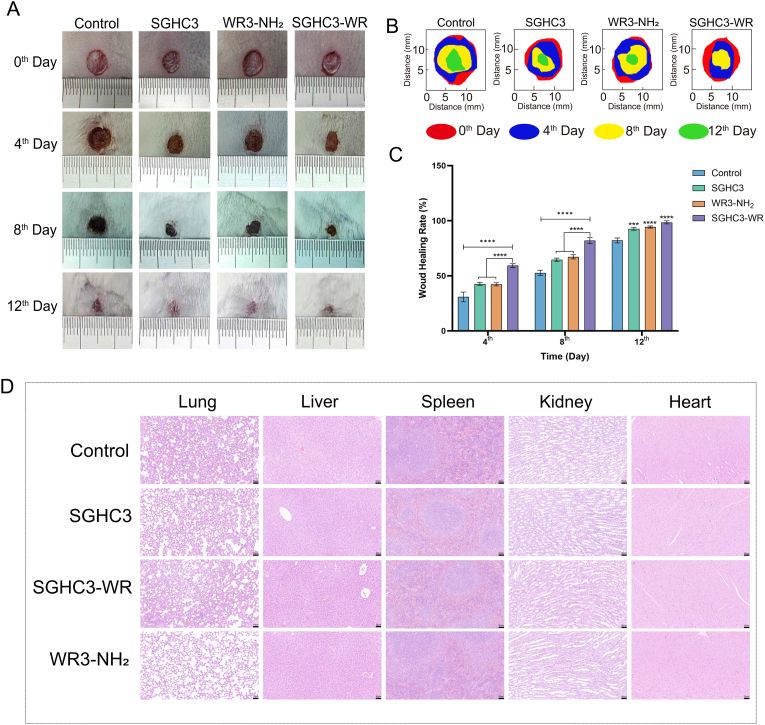
**Effects of SGHC3/SGHC3-WR and WR3-NH_2_ on the mouse full-thickness skin wound model. A**: Photographs of mouse wound repair; **B**: Simulation of mouse wound repair process; **C**: Wound healing rate of mice. **D**: Toxicity analysis of SGHC3/SGHC3-WR hydrogel on mouse organs. Error bars in the figures represent the mean standard error (SEM) from experiments with more than three replicates. Statistical significance is indicated as ∗∗∗∗ (p < 0.0001), ∗∗∗ (p < 0.001), ∗∗ (p < 0.01), ∗ (p < 0.05).

Skin wound healing is a complex physiological process that involves the crosstalk among various cells and signaling molecules [40,41]. In this study, a mouse full-thickness skin wound model was established to investigate the wound healing role of the prepared SGHC3 and SGHC3-WR hydrogels. Fig. 6A shows the wound recovery of the mice, which was documented using a smartphone camera. The wounds in the Control group healed slowly during the first four days, with a healing rate of only 30.88 %. In contrast, the healing rates for the SGHC3, WR3-NH_2_, and SGHC3-WR groups were 42.69 %, 42.30 %, and 59.31 %, respectively. The first week following skin wounding corresponds to the inflammatory phase [42,43]. In this study, WR3-NH_2_ exhibits certain anti-inflammatory properties, and the prepared hydrogel provides a moist environment conducive to wound repair while isolating the wound from environmental contamination. Thus, these factors contribute to the enhanced wound healing rates observed in the SGHC3, WR3-NH_2_ and SGHC3-WR groups compared to the Control group. Also, we have elaborated on the gelling mechanism of the SGHC3-WR hydrogel, and confirmed that WR3-NH_2_ does not undergo chemical crosslinking with the SGHC hydrogel, allowing for the release of WR3-NH_2_ under natural conditions (Fig. 3). Thus, the SGHC3 hydrogel and WR3-NH_2_ work synergistically to promote wound healing in mice, achieving a "1 + 1>2″ effect, which explains the rapid wound recovery in the SGHC3-WR group. By day 12, the scabs in the SGHC3 and WR3-NH_2_ groups had completely shed, while a small piece of scab remained in the Control group. Notably, the wounds in the SGHC3-WR group exhibited nearly complete recovery, with scab formation occurring earlier than that in the other three groups. This observation is supported by the absence of erythematous areas in the other groups, which may be attributed to the SGHC3-WR hydrogel's ability to accelerate wound remodeling in mice, thereby promoting a faster recovery. Fig. 6B presents a schematic diagram of the mouse wound repair process, visually illustrating the temporal changes in wounds across different groups. Fig. 6C displays the wound healing rates of mice in each group. Except on day 12, the healing rate of the SGHC3-WR group was significantly higher than those of the SGHC3 and WR3-NH_2_ groups. To ensure the safety, H&E staining of the mice internal organs were performed. The results (Fig. 6D) showed that the mice did not show any lesions in the heart, liver, spleen, lungs, and kidneys in either the SGHC3, SGHC3-WR, or WR3-NH_2_ groups, and there was no significant difference from the control group. This indicated that the hydrogels were safe and non-toxic. In summary, the prepared SGHC3 hydrogel effectively promotes full-thickness skin wound healing in mice, while the SGHC3-WR hydrogel loaded with AMPs, demonstrates enhanced efficacy in promoting wound healing.

### 3.7 Biological mechanism analysis of SGHC-WR hydrogel for wound healing

**Fig. 7:**
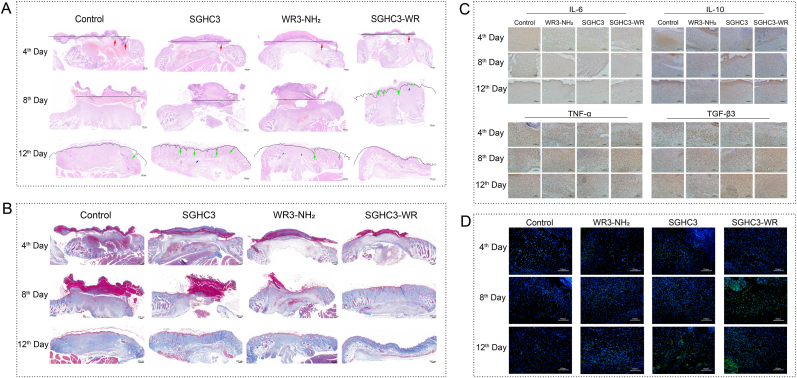
**Biological mechanism analysis of SGHC-WR hydrogel for wound healing. A:** H&E staining of mouse wound tissue. The black solid line indicates the wound size. The red arrow indicates inflammation. The green arrow indicates the hair follicle. The blue arrow indicates the blood vessel. **B**: Masson staining of mouse wound tissue. **C:** Immunohistochemistry of mouse wound tissue. **D**: CD31 immunofluorescence analysis of mouse wound tissue. (For interpretation of the references to colour in this figure legend, the reader is referred to the Web version of this article.)

During the investigation of SGHC3 and SGHC3-WR hydrogels on whole skin wound in mice, we found these hydrogels significantly enhanced wound healing and mitigated inflammatory responses. SGHC3 and SGHC3-WR hydrogels promoted histiocyte proliferation and increased scab thickness, as evidenced by H&E staining analysis (Fig. 7A). Notably, the SGHC3-WR group exhibited a faster healing rate with a concomitant reduction in inflammatory response. On day 8, neovascularization and hair follicles began to emerge in the WR3-NH_2_ group; conversely, in the SGHC3-WR group, the scabs had detached, and significant collagen fiber formation was observed. By day 12, scabs in all treatment groups had completely fallen off, revealing a fully formed epidermal layer, with hair follicles and blood vessels in the SGHC3-WR group appearing well-organized and resembling normal skin. Masson staining (Fig. 7B) further illustrated that while crusting began on day 4, collagen and muscle fiber proliferation was not yet significant. By day 8, the SGHC3-WR group displayed a notable increase and alignment of collagen fibers compared to the muscle fibers in the control group and other treatment groups. This suggests that the SGHC3-WR hydrogel may alleviate inflammation-induced tissue overgrowth and promote the proper alignment of collagen fibers, thereby reducing the likelihood of scar formation. The results of immunohistochemical analyses (Fig. 7C) indicates that SGHC3 and SGHC3-WR hydrogels may mitigate the inflammatory response by enhancing the expression of the anti-inflammatory factor IL-10. In comparison to the control group, WR3-NH_2_ and SGHC3-WR significantly promoted the secretion of the anti-scarring factor TGF-β3, thereby reinforcing their beneficial role in wound healing. Angiogenesis within tissues can be assessed and quantified through CD31 immunofluorescence staining. This assessment is crucial for studying neovascularization during the wound healing process, as effective angiogenesis enhances blood supply and supplies regenerating tissues with essential oxygen and nutrients, thereby expediting wound healing. CD31 immunofluorescence analysis (Fig. 7D) demonstrated that the SGHC3-WR hydrogel significantly increased the expression of CD31 on day 8, indicating its effectiveness in promoting neovascularization. This process not only facilitates the rapid repair of damaged tissues but may also diminish scar formation by improving the local microenvironment. In summary, the SGHC3-WR hydrogel creates a favorable environment for wound healing by reducing excessive inflammation, promoting epithelial formation and tissue remodeling, enhancing ordered collagen deposition, modulating key cytokines, and significantly promoting angiogenesis. These mechanisms not only accelerate the rate of wound healing but also enhance the quality of healing and reduce the likelihood of scar formation, underscoring its significant potential in wound therapy.

## 4 Summary and perspective

This study presents the successful development and systematic evaluation of a multifunctional hydrogel (SGHC-WR) that incorporates a novel AMP WR3-NH_2_ into a natural polysaccharide matrix to enhance wound healing applications. By synergizing the biological activity of WR3-NH_2_ with the structural and physicochemical advantages of the hydrogel, a biomaterial capable of addressing multiple challenges inherent in wound management was established, including infection control, inflammation regulation, tissue regeneration, and biosafety. At the cellular level, WR3-NH_2_ exhibited strong pro-healing activity by stimulating the proliferation and migration of HUVECs, HFF, and HaCaT. Importantly, WR3-NH_2_ effectively regulated cytokine secretion and modulated the MAPK signaling pathway, reducing the expression of pro-inflammatory mediators while enhancing anti-inflammatory and pro-regenerative factors such as IL-10 and TGF-β3. These findings underscore the dual antimicrobial and immunomodulatory functions of WR3-NH_2_, establishing it as a valuable therapeutic component for wound healing. Incorporating WR3-NH_2_ into the polysaccharide-based hydrogel allowed it to retain its bioactivity while enhancing stability and enabling controlled release. The SGHC-WR hydrogel demonstrated favorable physicochemical properties, including elasticity, injectability, porosity, and biodegradability. *In vitro* assays confirmed its broad-spectrum antimicrobial activity against both Gram-positive and Gram-negative bacteria, including multidrug-resistant strains. Furthermore, the hydrogel exhibited low hemolysis rates, excellent cytocompatibility, and no evidence of dermal irritation, thereby ensuring biosafety. *In vivo* experiments utilizing a full-thickness skin wound model further validated the therapeutic potential of SGHC-WR. Compared to controls, SGHC-WR accelerated wound closure, promoted neovascularization, and facilitated collagen deposition and tissue remodeling. Histological analyses revealed the regeneration of a well-structured epidermis, hair follicles, and blood vessels, indicating high-quality wound healing. Additionally, biosafety evaluations confirmed the absence of pathological damage in major organs, supporting the hydrogel's safety for clinical use. The hydrogel also demonstrated stable antimicrobial activity during long-term storage, which is a critical requirement for practical medical applications.

This work provides compelling evidence that SGHC-WR is a multifunctional wound dressing capable of integrating antimicrobial, anti-inflammatory, pro-healing, and hemostatic effects within a single platform. The synergistic interactions between WR3-NH_2_ and the polysaccharide hydrogel foster a favorable wound microenvironment, thereby accelerating repair while ensuring biocompatibility and stability. In conclusion, SGHC-WR represents a promising next-generation wound dressing with significant potential for clinical translation. Furthermore, the design principles demonstrated here may be extended to other biomedical applications requiring multifunctional biomaterials, such as tissue engineering and regenerative therapies. This study not only enhances the understanding of the synergy between AMPs and hydrogels but also lays a foundation for the rational design of safe and effective biomaterials for clinical wound management.

## CRediT authorship contribution statement

**Zhizhi Chen:** Writing – original draft, Methodology, Investigation, Data curation. **Chao Li:** Supervision, Project administration, Funding acquisition. **Lei Wang:** Methodology, Formal analysis, Data curation. **Ying Luo:** Supervision, Conceptualization. **Yahan Yang:** Resources. **Qinqin Han:** Software, Resources. **Jinyang Zhang:** Supervision, Software. **Yaoqiang Shi:** Writing – review & editing, Writing – original draft, Resources, Investigation. **Yi Sun:** Writing – review & editing, Supervision, Software, Resources. **Yuzhu Song:** Writing – review & editing, Project administration, Funding acquisition.

## Ethics approval statement

All animal experiments in this study were approved by the Animal Ethics Committee of Kunming University of Science and Technology (approval number: PZWH K2024-0008).

## Declaration of competing interest

The authors declare that they have no known competing financial interests or personal relationships that could have appeared to influence the work reported in this paper.

## Data Availability

Data will be made available on request.
